# A predictive nomogram for in-ICU deterioration of stage 1 pressure injuries: a retrospective study

**DOI:** 10.3389/fmed.2026.1835220

**Published:** 2026-05-18

**Authors:** Chenyan Zhang, Zhouzhou Dong, Wei Wang, Chensong Chen, Huina Xu

**Affiliations:** 1 Department of Intensive Care Unit, The Affiliated Lihuili Hospital of Ningbo University, Ningbo, China; 2 Xiangshan First People's Hospital Medical and Health Group, Ningbo, China

**Keywords:** critical care, intensive care unit, nomogram, prediction model, pressure injury

## Abstract

**Background:**

Preventing Stage 1 pressure injuries (PIs) from worsening in the ICU is a key clinical challenge. Early prediction of high-risk patients enables targeted prevention. We aimed to develop a model for this progression using admission data.

**Methods:**

In this retrospective cohort study, eligible ICU patients with Stage 1 pressure injuries were randomly allocated into training (70%) and validation (30%) sets. Predictors were selected using LASSO regression. A multivariable logistic regression model was constructed and visualized as a nomogram. Model performance was evaluated by discrimination (AUC), calibration, and clinical utility (decision curve analysis).

**Results:**

A total of 278 patients were randomly divided into training (*n* = 195) and validation (*n* = 83) sets. LASSO regression identified three independent predictors: diabetes (OR: 2.451; 95% CI: 1.139–5.274), maximum norepinephrine dose (OR: 2.051; 95% CI: 1.322–3.182), and albumin level at ICU admission (OR: 0.834 per unit increase; 95% CI: 0.776–0.897). The nomogram demonstrated excellent discrimination, with an AUC of 0.800 (95% CI: 0.736–0.865) in the training set and 0.785 (95% CI: 0.675–0.895) in the validation set. Good calibration and clinical utility were confirmed.

**Conclusions:**

A nomogram incorporating three readily available factors at ICU admission effectively predicts the risk of Stage 1 PI progression. This tool is designed to aid early risk stratification and could help guide preventive measures.

## Introduction

Pressure injuries (PIs) remain a prevalent and costly complication in ICU patients, contributing to extended hospitalization and increased healthcare costs (1, 2). Despite established prevention protocols, the incidence of PIs in intensive care settings persists at concerning levels (3). A particularly challenging clinical scenario is the management of Stage 1 PIs. Presenting as non-blanchable erythema, they signify the earliest point of detectable tissue compromise, representing a critical window for intervention where damage is potentially reversible (4).

The core clinical dilemma stems from the highly variable natural history of these early lesions; not all Stage 1 PIs progress to more severe, full-thickness wounds (≥Stage 2) (4). This variability makes it exceedingly difficult to distinguish, at the bedside, which patients are on a trajectory of rapid deterioration. Current generalized risk assessment tools, while valuable for screening, demonstrate limited accuracy in predicting the specific outcome of progression from an existing Stage 1 injury within the complex ICU environment (5). These tools, such as the Braden Scale, were primarily developed and validated for predicting the *incidence* of new pressure injuries in broader populations. Their performance in discerning which early, established lesions (Stage 1) will deteriorate rapidly in critically ill patients remains suboptimal. This predictive uncertainty directly contributes to suboptimal prevention strategies, leading to either inefficient resource allocation or missed opportunities for timely, intensified care (6).

An accurate predictive model using data readily available at admission could fundamentally shift this paradigm by enabling true early risk stratification (7). Such a tool would allow clinicians to preemptively identify high-risk individuals, facilitating precise interventions before irreversible tissue damage occurs (8). Although predictive models have been successfully applied to forecast other critical outcomes (9), a specific, validated tool designed to estimate the individual risk of Stage 1 PI progression remains an unmet clinical need (10).

Therefore, the primary objective of this study was to develop and validate a novel prediction model, visualized as a clinically practical nomogram. This tool aims to individually estimate the risk of progression from Stage 1 to severe PIs (≥Stage 2) in ICU patients using only data that is routinely collected within the first 24 h of admission.

## Materials and methods

### Study population

**Figure 1:**
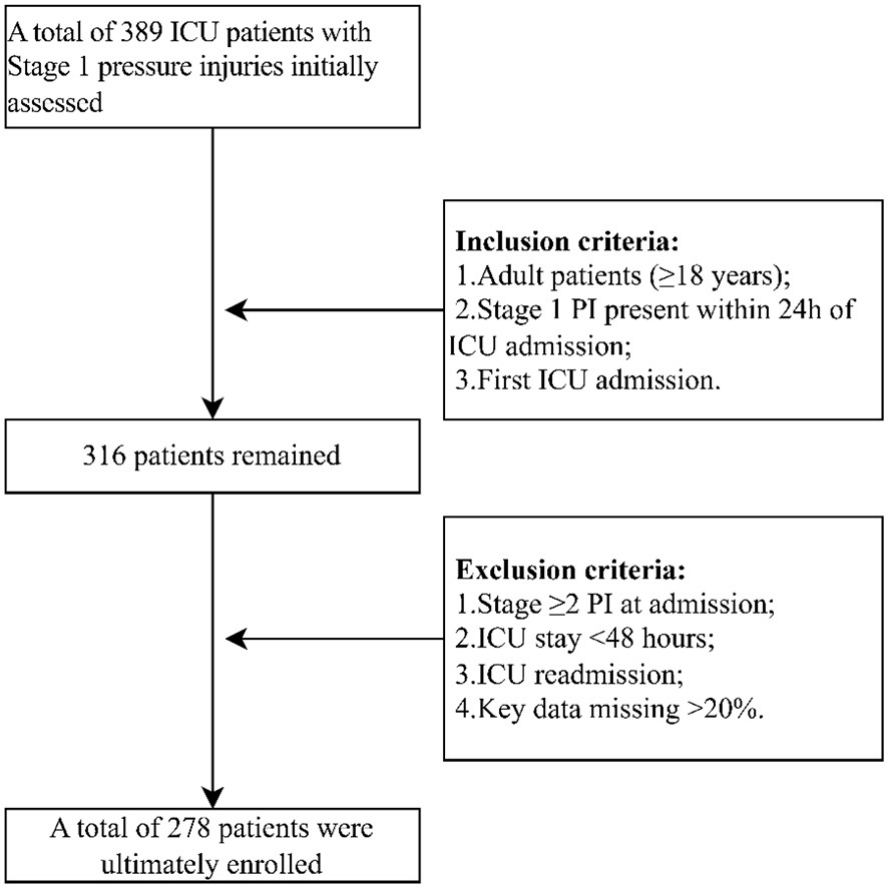
Flowchart of participant screening and enrollment in this cohort study; PI, pressure injury; ICU, intensive care unit.

This single-center, retrospective cohort study was conducted at The Affiliated Lihuili Hospital of Ningbo University. This study was reported in accordance with the Transparent Reporting of a Multivariable Prediction Model for Individual Prognosis or Diagnosis (TRIPOD) statement. The completed checklist is provided as Supplementary material 1. The detailed patient screening process is shown in Figure 1. Patients were included if they met the following criteria: (1) adult patients (≥18 years) admitted to the ICU between January 2022 and December 2025; (2) presence of at least one Stage 1 pressure injury (PI) identified within 24 h of ICU admission; (3) first ICU admission during the current hospitalization. The diagnosis and staging of PIs were performed by trained ICU nurses according to the EPUAP/NPIAP/PPPIA 2019 International Clinical Practice Guideline (11). Skin assessments were conducted at least every 8–12 h during routine care. Stage 1 PI (non-blanchable erythema) was distinguished from blanchable erythema, moisture-associated skin damage, and incontinence-associated dermatitis primarily via finger pressure test and assessment of skin integrity. In cases of ambiguous documentation, a senior study nurse with wound care certification reviewed all relevant records to adjudicate the final stage. To ensure complete outcome ascertainment, the final study database was established and locked on February 1, 2026. This process guaranteed that the primary outcome (progression or non-progression of the Stage 1 PI) was definitively determined for all patients, including those admitted at the end of the recruitment period. The exclusion criteria were as follows: (1) patients with Stage 2 or higher PIs at ICU admission; (2) patients with an ICU length of stay < 48 h; (3) patients who were readmitted to the ICU during the same hospitalization; (4) patients with major missing clinical data (>20% of key variables).

This study was approved by the Medical Ethics Committee of The Affiliated Lihuili Hospital of Ningbo University (Approval No. IIT2026SL0013). The requirement for informed consent was waived due to the retrospective nature of the study. All procedures were conducted in accordance with the Declaration of Helsinki.

### Grouping and data collection

This study aimed to develop a prediction model for the progression of Stage 1 pressure injuries (PIs). Therefore, the study population was categorized into two groups based on the primary outcome: the progression group (patients whose Stage 1 PIs worsened to Stage 2 or beyond during their ICU stay) and the non-progression group (patients whose Stage 1 PIs did not worsen). This grouping allowed for a direct comparison of factors associated with the key clinical event.

Data pertaining to demographic characteristics, disease severity scores, laboratory parameters at ICU admission, and details of treatment interventions were retrospectively extracted from the hospital's electronic medical record system. To ensure the model's utility for early risk stratification, data collection was strictly focused on variables that are routinely available within the first 24 h of ICU admission, such as APACHE II score, serum albumin level, and the use of critical care supports like mechanical ventilation and vasoactive drugs. The three predictors in the final model were defined as follows: diabetes (pre-existing history), maximum norepinephrine dose (highest rate in μg/kg/min during the first 24 h), and serum albumin (first measurement after ICU admission).

### Statistical analysis

Continuous variables not conforming to a normal distribution were presented as medians with interquartile ranges (IQR) and compared using the Mann–Whitney *U* test. Categorical variables were expressed as counts and percentages, with between-group comparisons made using the chi-square test. The entire cohort was randomly divided into a training set and a validation set at a ratio of 7:3. We employed a hierarchical internal validation strategy. The primary framework was this 70/30 random split, which provided an independent validation set for the most direct estimate of performance on unseen data. To obtain robust, optimism-corrected performance estimates and their confidence intervals, bootstrap resampling (*B* = 1,000) was applied separately to both the training and validation sets. Additionally, ten-fold cross-validation was conducted on the training set as a supplementary procedure to assess the model's stability across different data subsets.

Independent predictors were selected through Least Absolute Shrinkage and Selection Operator (LASSO) regression. A multivariable logistic regression model was subsequently constructed, and a nomogram was developed using the “rms” package in R. The predictive performance of the nomogram was evaluated by its discrimination (assessed using the receiver operating characteristic curve and quantified by the area under the curve), calibration (assessed using calibration curves), and clinical utility (assessed using decision curve analysis to estimate the net benefit across a range of threshold probabilities). A two-sided *p*-value < 0.05 was considered statistically significant. No data imputation was performed as the variables included in the final model (diabetes, maximum norepinephrine dose, serum albumin) had complete data. To assess the potential bias introduced by the use of complete-case analysis for other candidate variables, a sensitivity analysis comparing the three variables that contained any missing data (APACHE II score, duration of mechanical ventilation, and Braden score) was conducted between the training and validation sets; no significant differences were found, supporting the assumption that the missing data were not systematically different (Supplementary Table S2). Given the exploratory nature of this retrospective study, a formal *a priori* sample size calculation was not performed. We included all eligible patients meeting the study criteria during the defined time period. The effective sample size for model development and the number of outcome events are reported in the Results section to allow for assessment of model stability and the events-per-variable (EPV) ratio. A pre-specified sensitivity analysis was conducted to assess the robustness of the model under a more stringent outcome definition. In this analysis, the primary outcome was redefined as the development of a Stage 3 or higher pressure injury, or any full-thickness injury (i.e., encompassing Stage 3, Stage 4, unstageable, and deep tissue injury). The same candidate predictors and model-building procedures (LASSO regression followed by multivariable logistic regression) were applied to this revised endpoint.

To account for varying time at risk, a Cox proportional-hazards regression model was also employed, with the time from ICU admission to the first observation of severe PI progression as the outcome. Patients who were discharged from the ICU or died without experiencing PI progression were right-censored at their ICU discharge or death time, respectively. The proportional hazards assumption was checked and satisfied. To formally account for death and discharge as competing events, a Fine-Gray competing-risks regression was performed. Additionally, as a sensitivity analysis to directly assess the potential confounding effect of mortality, a standard Cox proportional hazards model was applied to the subset of patients who survived the ICU stay (i.e., excluding those who died). The results of this analysis are presented separately. Furthermore, to address potential concerns regarding sparse data for the continuous maximum norepinephrine dose variable, a sensitivity analysis using Firth's penalized logistic regression was performed, confirming the stability of its estimated effect (OR = 2.00, 95% CI: 1.32–3.14). The detailed results of this analysis are provided in Supplementary Table S3.

## Results

### Patient characteristics and univariate analysis

A total of 278 eligible ICU patients with Stage 1 PIs were enrolled in this study. The cohort was randomly divided into a training set (*n* = 195, 70%) and a validation set (*n* = 83, 30%). Within the training set, 133 patients (68.2%) experienced the primary outcome (progression to severe PI). The initial LASSO regression screened 21 candidate variables, yielding an EPV of 6.3 (133 events/21 variables) for the variable selection phase. In the final 3-predictor model, the EPV was 44.3 (133 events/3 predictors), which exceeds common recommendations for logistic regression model stability. As detailed in Supplementary Table S1, no statistically significant differences (all *P* > 0.05) were observed between the training and validation sets across all baseline characteristics, including demographics, clinical severity scores, comorbidities, and laboratory parameters. This indicates a successful and balanced randomization, ensuring the comparability of the two datasets for subsequent model development and validation.

Table 1 presents a comparison of baseline characteristics between patients with non-severe and severe pressure injuries (PIs) in the training set. Significant differences were observed between the two groups. The severe PI group had a higher prevalence of diabetes (43.61% vs. 24.19%, *P* = 0.009), required a greater maximum norepinephrine dose (median 0.08 vs. 0 μg/kg/min, *P* = 0.047), and exhibited a significantly lower serum albumin level at ICU admission (26.89 g/L vs. 31.81 g/L, *P* < 0.001). No statistically significant differences were found in other demographic, clinical, or laboratory parameters.

**Table 1:** Comparison of baseline characteristics between non-severe and severe pressure injury groups in the training set.

Variable	Non-severe (*n* = 62)	Severe (*n* = 133)	*X*^2^/*t*/*z*	*P*-value
Age (years)	72.11 ± 15.3	72.7 ± 13.83	−0.266	0.790
**Gender**, ***n*** **(%)**				
Male	45 (72.58)	99 (74.44)	0.075	0.784
Female	17 (27.42)	34 (25.56)		
BMI (kg/m^2^)	21.78 ± 2.90	21.81 ± 3.41	−0.066	0.948
**Infection site**, ***n*** **(%)**				
Pulmonary	37 (59.68)	96 (72.18)	5.239	0.155
Abdominal	4 (6.45)	12 (9.02)		
Bloodstream	0 (0.00)	0 (0.00)		
Urinary system	3 (4.84)	3 (2.26)		
Others	18 (29.03)	22 (16.54)		
**Most severe PI site**, ***n*** **(%)**				
Supine position group	51 (82.26)	94 (70.68)	3.014	0.222
Lateral/positioning group	9 (14.52)	33 (24.81)		
Device-related group	2 (3.23)	6 (4.51)		
APACHE II at ICU admission	17 (13, 21)	17 (14, 22)	−0.605	0.546
**Diabetes**, ***n*** **(%)**				
No	47 (75.81)	75 (56.39)	6.806	0.009
Yes	15 (24.19)	58 (43.61)		
Hospital stay (days)	25.5 (16, 37)	22 (12, 31)	−1.641	0.101
Lactate (mmol/L)	1.7 (1.2, 2.5)	1.6 (1.3, 2.4)	−0.162	0.871
**Septic shock**, ***n*** **(%)**				
No	44 (70.97)	82 (61.65)	1.604	0.205
Yes	18 (29.03)	51 (38.35)		
**Vasopressor use**, ***n*** **(%)**				
No	32 (51.61)	53 (39.85)	2.380	0.123
Yes	30 (48.39)	80 (60.15)		
**Max norepinephrine dose (**μ**g/kg/min)**				
< 0.1μg/kg/min	43 (69.35)	54 (40.6)	14.126	0.001
0.1–0.3μg/kg/min	9 (14.52)	33 (24.81)		
>0.3 μg/kg/min	10 (16.13)	46 (34.59)		
**Mechanical ventilation**, ***n*** **(%)**				
No	14 (22.58)	19 (14.29)	2.070	0.150
Yes	48 (77.42)	114 (85.71)		
Mechanical ventilation duration (days)	5.5 (2,11)	7 (3,14)	−1.176	0.240
**Deep sedation**, ***n*** **(%)**				
No	45 (72.58)	86 (64.66)	1.203	0.273
Yes	17 (27.42)	47 (35.34)		
Fluid balance on ICU day 1 (ml)	1094 (599, 1,779)	820 (175, 1,583)	−1.807	0.071
Braden score at ICU admission	10 (10, 11)	10 (10, 11)	−1.824	0.068
**Braden moisture subscore**, ***n*** **(%)**				
1	0 (0.00)	1 (0.75)	3.379	0.337
2	9 (14.52)	32 (24.06)		
3	46 (74.19)	85 (63.91)		
4	7 (11.29)	15 (11.28)		
**Skin moisture management**, ***n*** **(%)**				
No	49 (79.03)	100 (75.19)	0.347	0.556
Yes	13 (20.97)	33 (24.81)		
Avg. enteral nutrition intake (first 3 days) (kcal/day)	0 (0, 18)	8 (0, 17.45)	−1.126	0.260
Albumin at ICU admission (g/L)	31.81 ± 5.63	26.89 ± 5.24	5.958	< 0.001

PI, pressure injury; BMI, body mass index; APACHE II, Acute Physiology and Chronic Health Evaluation II; ICU, intensive care unit.

### Predictor selection and model construction

**Figure 2:**
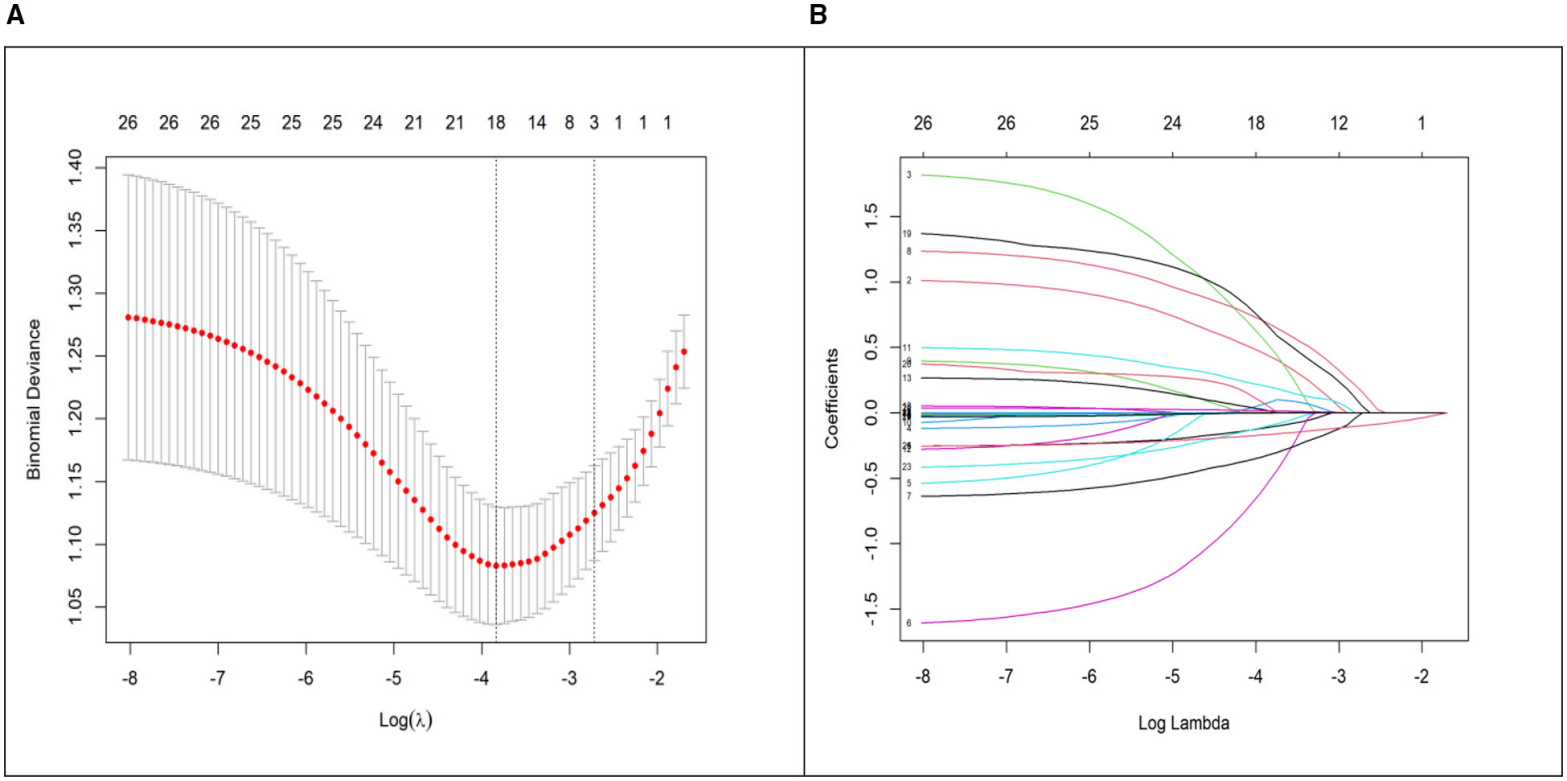
LASSO-logistic regression results. **(A)** Cross-validation plot. **(B)** Selection process by cross-validation method.

Variable selection was performed using Least Absolute Shrinkage and Selection Operator (LASSO) regression with ten-fold cross-validation. LASSO regression was employed for its ability to handle correlated predictors and perform automatic variable selection through coefficient shrinkage, thereby reducing the risk of over fitting in the initial screening phase from the 21 candidate variables listed in Table 1. The optimal lambda value (λ = 0.0660) was determined as the lambda.1se, which yielded three predictors with non-zero coefficients: diabetes, maximum norepinephrine dose, and albumin level at ICU admission (Figure 2). These selected variables were then entered into a standard multivariable logistic regression model. This subsequent step provides easily interpretable odds ratios and confidence intervals, which are essential for clinical understanding and for constructing the practical nomogram. As presented in Table 2, all of them were identified as independent predictors for severe pressure injuries: diabetes, maximum norepinephrine dose, and albumin level at ICU admission.

**Table 2:** Multivariable logistic regression analysis for independent predictors of severe pressure injury.

Variable	*B*	S.E.	Wald	*P*	OR	95%CI		Collinearity diagnostics	
						LL	UL	Tolerance	VIF
Diabetes	0.897	0.391	5.259	0.022	2.451	1.139	5.274	0.995	1.005
Maximum norepinephrine dose	0.718	0.224	10.263	0.001	2.051	1.322	3.182	0.982	1.018
Albumin at ICU admission	−0.181	0.037	24.094	< 0.001	0.834	0.776	0.897	0.985	1.015
Constant	4.56	1.117	16.666	0.000	95.614				

B, regression coefficient; S.E., standard error; Wald, Wald statistic; *P, P*-value; OR, odds ratio; CI, confidence interval; LL, lower limit; UL, upper limit; VIF, variance inflation factor; ICU, intensive care unit.

**Figure 3:**
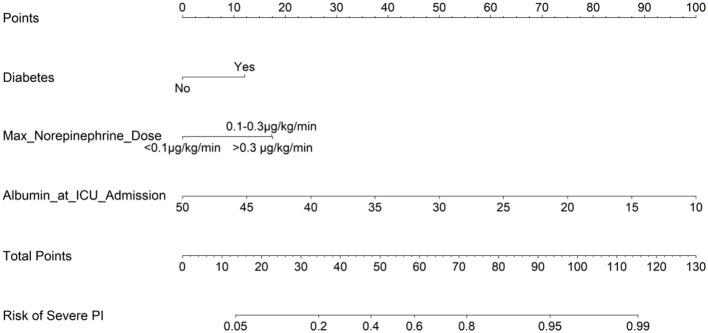
Nomogram for predicting the risk of severe pressure injury in ICU patients with Stage 1 injury. The nomogram incorporates three predictors: diabetes (yes/no), maximum norepinephrine dose (μg/kg/min), and serum albumin level at ICU admission (g/L). To use the nomogram, locate the patient's value on each variable axis, draw a line upward to the “Points” axis to obtain the individual score, sum all scores to get the “Total Points”, and then draw a line downward from the “Total Points” axis to the bottom scale to read the predicted probability of severe PI (Stage 2 or above) progression.

Based on the three independent predictors identified in the multivariable logistic regression analysis (Table 2)—a history of diabetes, maximum norepinephrine dose, and serum albumin level at ICU admission—a nomogram was constructed to visualize the final prediction model (Figure 3). The nomogram provides a user-friendly tool for individualized risk estimation. Clinicians can obtain a patient's total points by summing the individual scores assigned to each predictor variable. This total score corresponds to a specific predicted probability on the bottom scale, representing the individual's risk of progressing from Stage 1 to severe PI (Stage 2 or above), thereby facilitating rapid bedside risk assessment.

### Practical illustration of nomogram use

**Figure 4:**
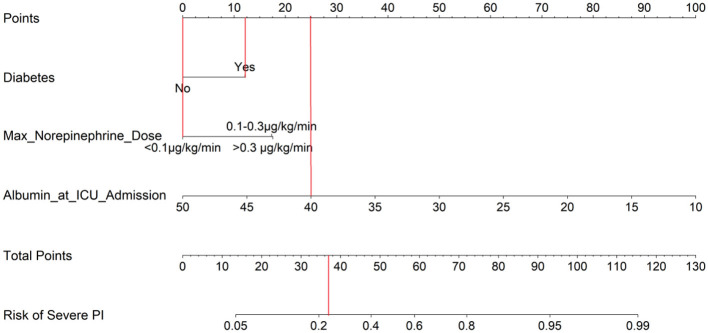
Nomogram for Predicting Progression from Stage 1 to Severe Pressure Injury in Critically Ill Patients

To demonstrate the clinical application of the nomogram, consider a representative patient with diabetes, a maximum norepinephrine dose of 0.05 μg/kg/min, and an admission albumin level of 40 g/L. Using the nomogram (Figure 4), this profile yields a total score of 37 points, corresponding to a predicted approximately 23% risk of progression to severe PI. This estimate, which falls within the model's actionable range, directly illustrates how the tool enables individualized risk stratification to guide intensified preventive measures at the bedside.

### Model performance and validation

**Figure 5:**
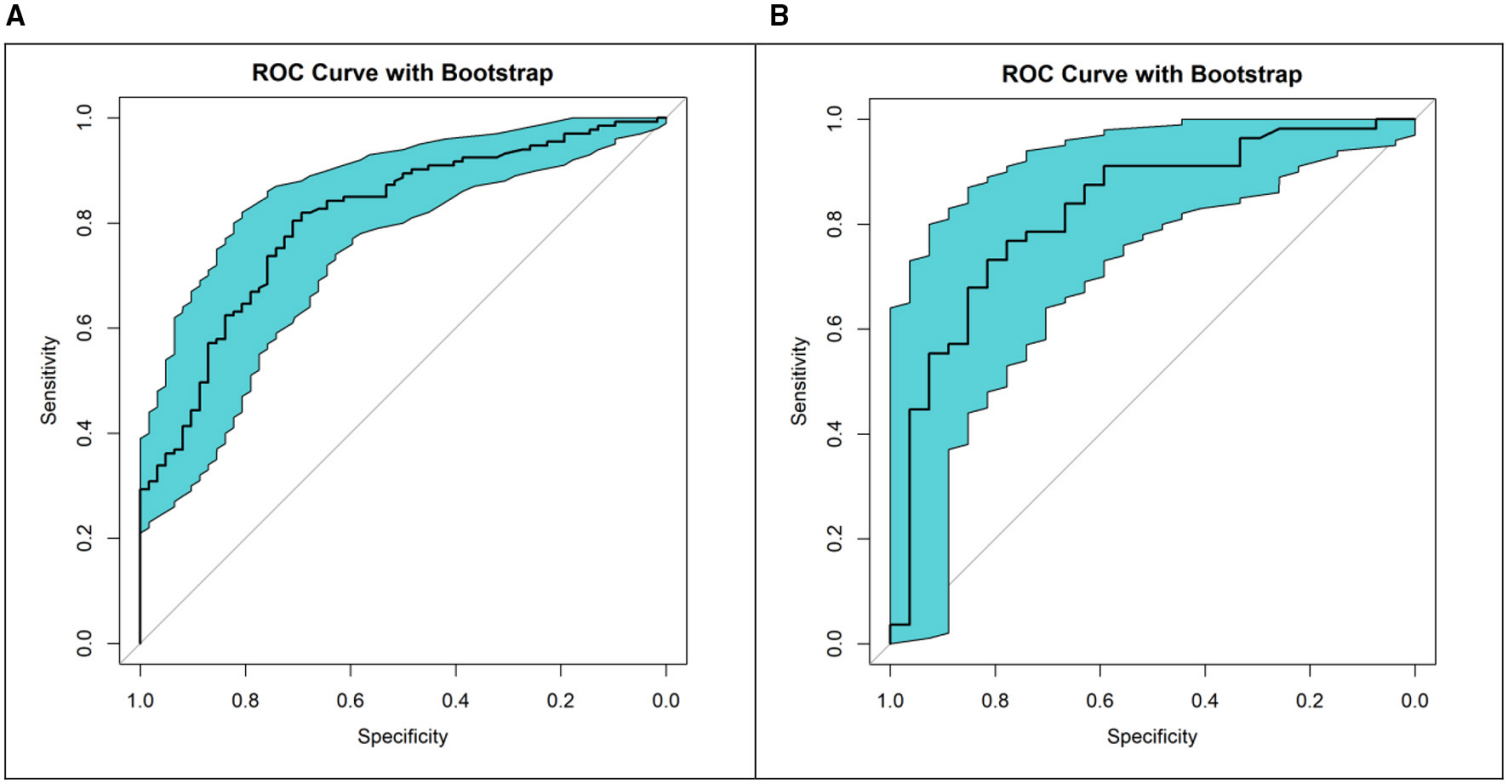
Bootstrap-enhanced receiver operating characteristic (ROC) curves of the nomogram for predicting severe pressure injury. **(A)** ROC curve in the training set. **(B)** ROC curve in the validation set.

The predictive performance of the nomogram was evaluated using bootstrap resampling (*n* = 1,000 iterations) applied to the training and validation sets, respectively, to obtain robust estimates and their variability. The bootstrap-derived receiver operating characteristic (ROC) analysis demonstrated good discriminatory ability. The area under the curve (AUC) was 0.800 (95% CI: 0.736–0.865) in the training set, with a sensitivity of 78.2%, specificity of 74.2%, positive predictive value (PPV) of 86.7%, negative predictive value (NPV) of 61.3%, and an overall diagnostic accuracy of 76.9%. In the validation set, the AUC was 0.785 (95% CI: 0.675–0.895), with sensitivity of 80.4%, specificity of 74.1%, PPV of 86.5%, NPV of 64.5%, and accuracy of 78.3% (Figure 5).

**Figure 6:**
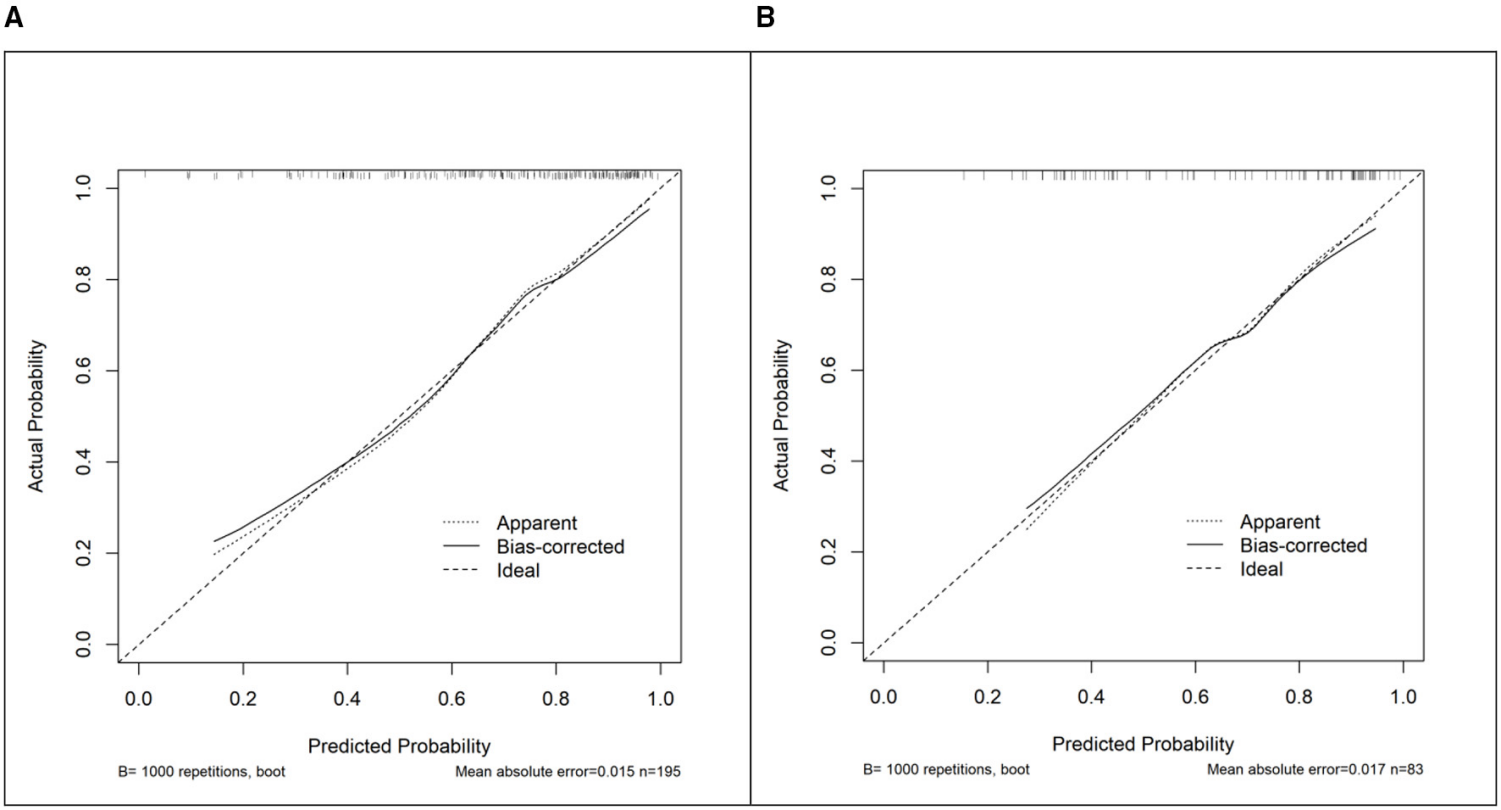
Calibration curves of the nomogram for predicting severe pressure injury. **(A)** Calibration curve in the training set. **(B)** Calibration curve in the validation set. *B* = 1,000 repetitions, boot, bootstrap repetitions.

Calibration analysis, also based on bootstrap resampling, revealed good model fit. The Hosmer–Lemeshow test results (training set: χ^2^=11.234, *P* = 0.189; validation set: χ^2^ = 10.240, *P* = 0.249) indicated no significant deviation. The calibration curves showed close alignment between predicted and observed probabilities. Key calibration metrics from the bootstrap analysis included a mean absolute error of 0.015, a calibration intercept of 0.018, a calibration slope of 0.987, and a Brier score of 0.163 in the training set. Corresponding values in the validation set were 0.017, −0.025, 0.961, and 0.155, respectively (Figure 6).

**Figure 7:**
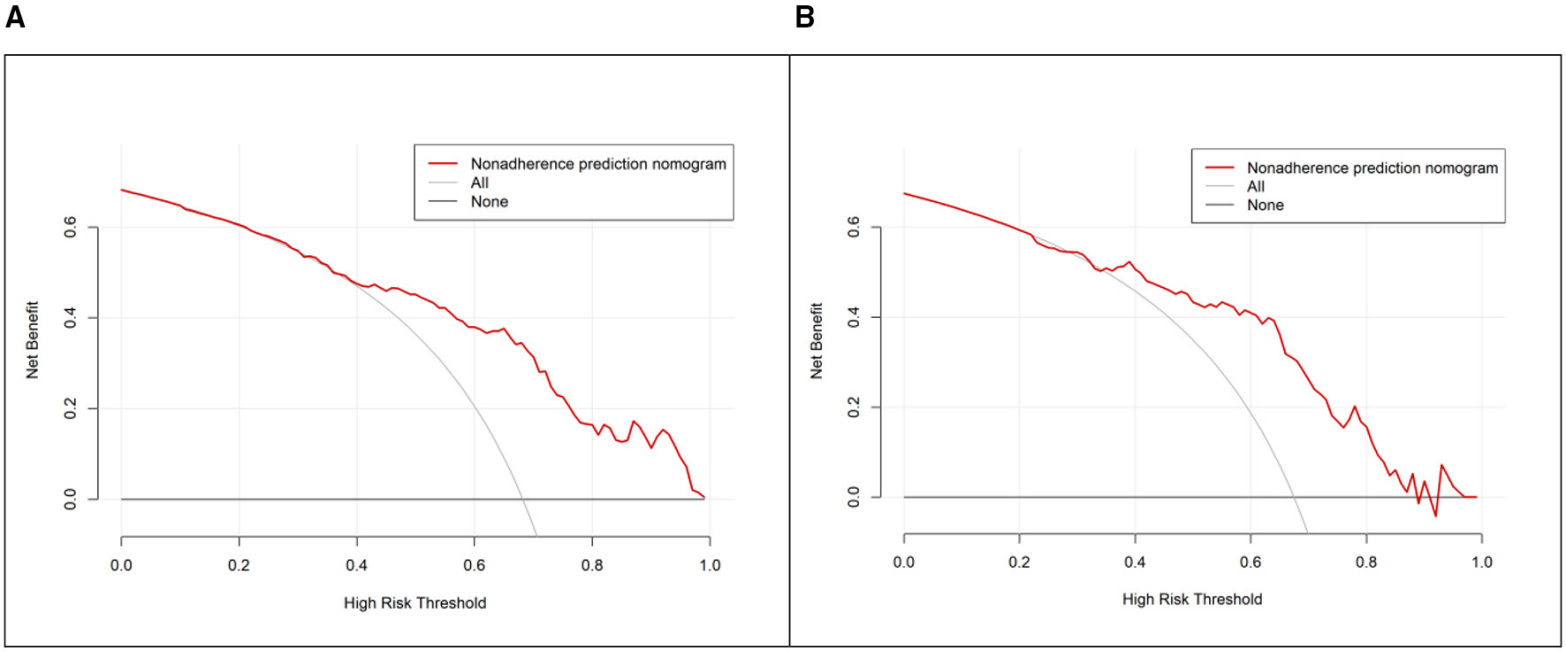
Decision curve analysis (DCA) of the nomogram for predicting severe pressure injury. **(A)** Net benefit curve in the training set. **(B)** Net benefit curve in the validation set.

Decision curve analysis (DCA) demonstrated the model's clinical utility. In the training set, the nomogram provided a positive net benefit over the “treat-all” and “treat-none” strategies across a wide range of high-risk thresholds (approximately 0.40–0.99). A similar trend was observed in the validation set, where the net benefit curve of the nomogram consistently remained above both reference curves, supporting its clinical applicability and generalizability (Figure 7).

**Figure 8:**
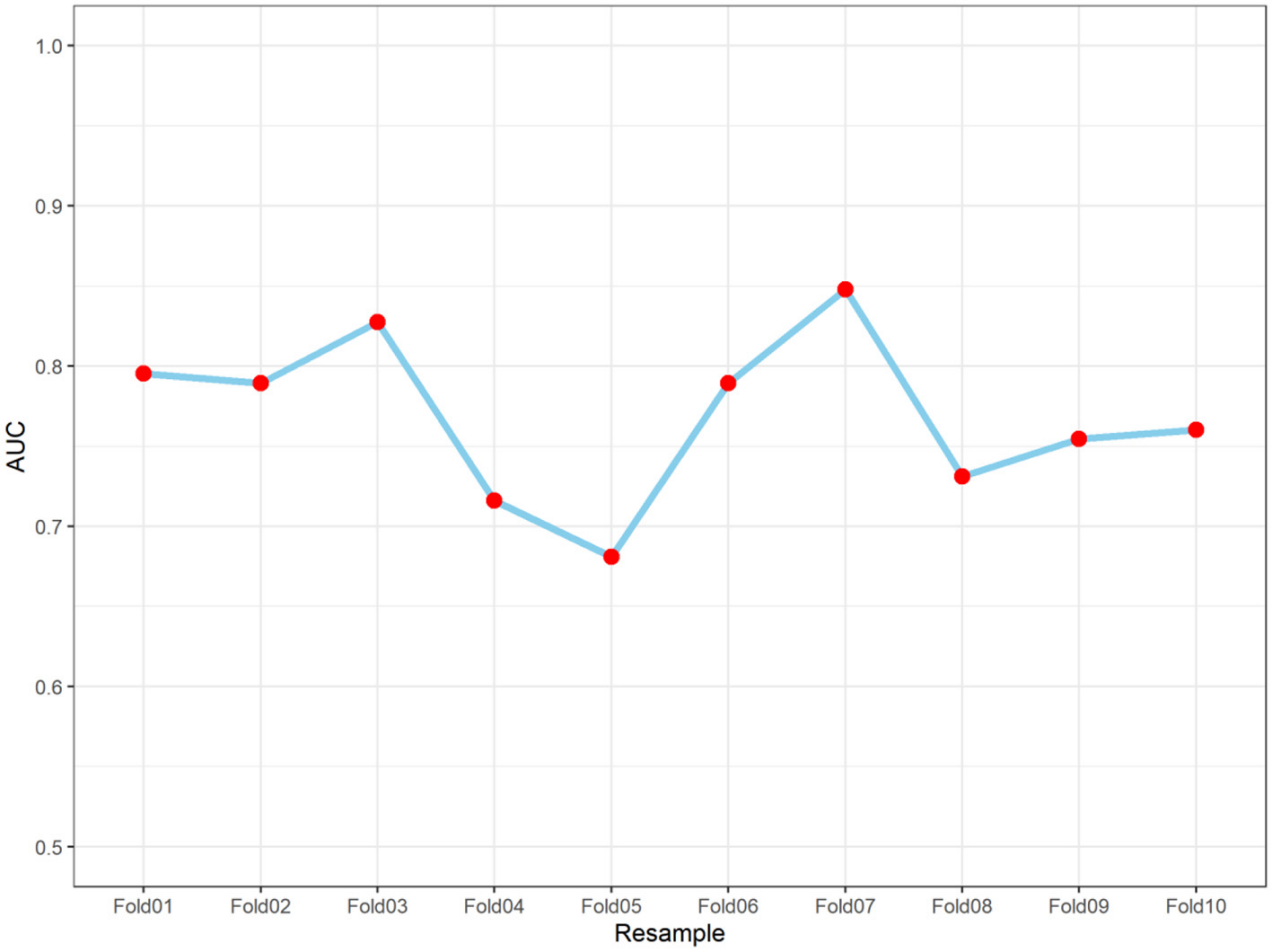
Performance of the nomogram for predicting severe pressure injury in ten-fold cross-validation. AUC, area under the curve.

To evaluate the model's performance and generalizability, ten-fold cross-validation was conducted. The above Figure 8 illustrates the accuracy of the model across each of the ten-folds (Fold01 to Fold10). The overall area under the curve (AUC) from the ten-fold cross-validation was 0.769 (95% confidence interval: 0.733–0.806), with values fluctuating relatively narrowly around 0.76. This indicates that the model exhibited consistent performance across different data subsets, demonstrating good stability without obvious overfitting or underfitting, and there were no significant fluctuations in performance among the folds. In summary, the model demonstrated favorable predictive performance and robustness in this study.

### Supplemental full-cohort validation

To further confirm the stability of our findings, we conducted bootstrap validation on the entire cohort (*n* = 278). As shown in Supplementary Figure 1, the optimism-corrected AUC remained consistent at 0.787 (95% CI: 0.731–0.843). The calibration plot (Supplementary Figure 2) indicated excellent agreement between predicted and observed probabilities, with a mean absolute error of 0.011, a Brier score of 0.170, a calibration intercept of −0.052, and a calibration slope of 0.971. Decision curve analysis (Supplementary Figure 3) further confirmed a significant net benefit across a broad range of risk thresholds (0.33–0.98), supporting the clinical utility of the model.

### Sensitivity analysis with a stricter outcome definition

To address the clinical heterogeneity within the original outcome definition, we performed a sensitivity analysis redefining severe PI as Stage 3 or above (full-thickness injury). Among the 189 patients in the entire cohort whose Stage 1 PIs progressed, the most severe injury stage was distributed as follows: 113 (59.8%) Stage 2, 53 (28.0%) Stage 3, 13 (6.9%) Stage 4, 6 (3.2%) unstageable, and 4 (2.1%) deep tissue injury. This distribution confirms that a substantial proportion (40.2%) progressed to full-thickness injury (Stage 3+), justifying a focused analysis on this more severe endpoint.

**Figure 9:**
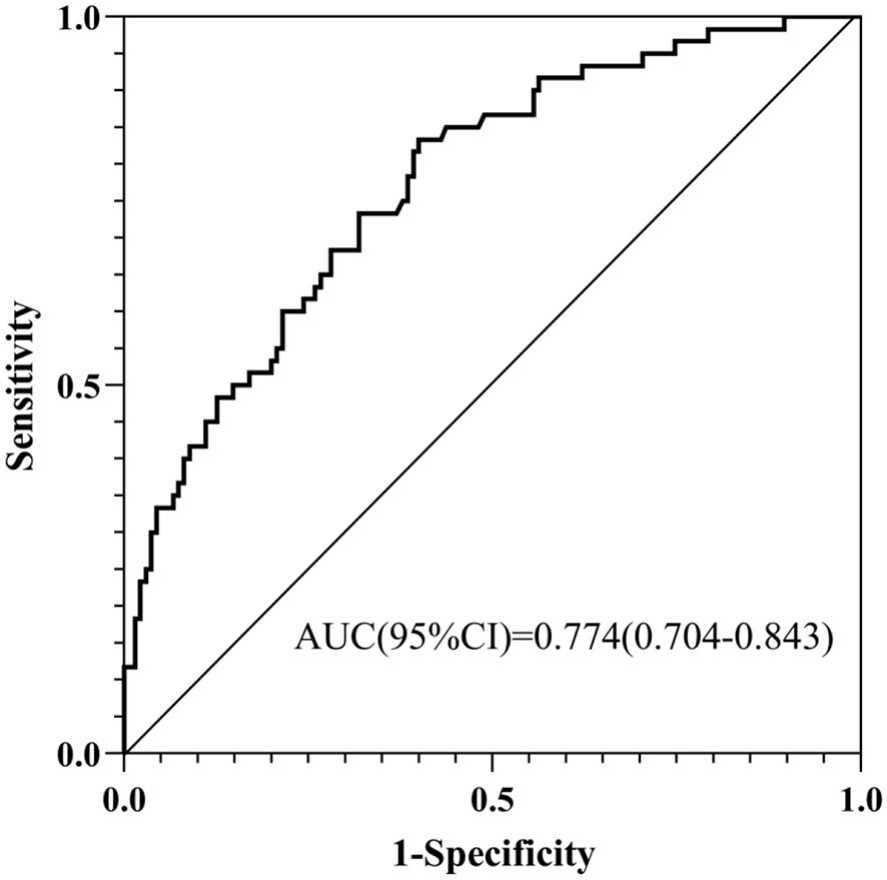
Receiver Operating Characteristic (ROC) curve for the prediction model of severe pressure injury (Stage 3+ or full-thickness injury).

Using the same three admission predictors (diabetes, maximum norepinephrine dose, and serum albumin), a new logistic regression model was fitted (Table 3). This model demonstrated good and consistent discriminatory ability, with an AUC of 0.774 (95% CI: 0.704–0.843) (Figure 9). The results indicate that all three identified predictors remain robust in forecasting the progression to more severe tissue damage.

**Table 3:** Multivariable logistic regression analysis for predictors of severe pressure injury (Stage 3+ or full-thickness injury).

Variable	*B*	SE	Wald	*P*	OR	95%CI	
						LL	UL
Diabetes	0.636	0.272	5.472	0.019	1.888	1.109	3.217
Maximum norepinephrine dose	0.697	0.160	19.001	< 0.001	2.007	1.467	2.745
Albumin at ICU admission	−0.063	0.025	6.048	0.014	0.939	0.894	0.987
Constant	−0.437	0.803	0.296	0.586	0.646		

### Cox regression and sensitivity analysis

To address the time-dependent nature of the outcome, a Cox proportional-hazards regression model was fitted. The model, presented in Table 4, confirmed that diabetes (HR = 1.403, 95% CI: 1.050–1.875), a higher maximum norepinephrine dose category (HR = 1.236, 95% CI: 1.040–1.469), and lower serum albumin at admission (HR = 0.929 per unit increase, 95% CI: 0.903–0.955) were independently associated with a shorter time to severe PI progression. This analysis is contextualized by the observed time-to-event data: the median time to progression in the progression group was 13 days, while the median ICU length of stay (representing the follow-up time at risk) in the non-progression group was 27 days. This temporal framework underscores the clinical relevance of the identified risk factors within the typical ICU admission period.

**Table 4:** Cox proportional hazards regression analysis for time to progression of pressure injury (unadjusted for competing risks).

Variable	*B*	SE	Wald	*P*	HR	95%CI	
						LL	UL
Diabetes	0.339	0.148	5.247	0.022	1.403	1.050	1.875
Maximum norepinephrine dose	0.212	0.088	5.790	0.016	1.236	1.040	1.469
Albumin at ICU admission	−0.074	0.014	27.168	< 0.001	0.929	0.903	0.955

To formally account for death and discharge as competing events, a Fine-Gray subdistribution proportional hazards regression was performed. The results, presented in Table 5, demonstrated that all three predictors (diabetes, maximum norepinephrine dose, and serum albumin) remained statistically significant predictors of the cumulative incidence of PI progression. The hazard ratios were directionally consistent and of similar magnitude to those from the primary Cox model (Table 4), reinforcing the robustness of the associations in the presence of competing risks.

**Table 5:** Fine-gray competing-risks regression (adjusted for ICU length of stay).

Variable	*B*	SE	Wald	*P*	HR	95%CI	
						LL	UL
Diabetes	0.452	0.151	8.963	0.003	1.571	1.169	2.111
Maximum norepinephrine dose	0.189	0.089	4.495	0.034	1.208	1.014	1.439
Albumin at ICU admission	−0.059	0.014	17.568	< 0.001	0.943	0.917	0.969

As a direct check for potential confounding by mortality, a sensitivity analysis was conducted using a Cox proportional hazards model on the subset of patients who survived the ICU stay. The results, presented in Table 6, were nearly identical to the primary analysis, with all three predictors remaining significant. This indicates that the identified associations are not substantially confounded by differential mortality between groups.

**Table 6:** Cox proportional hazards regression (excluding deaths, sensitivity analysis).

Variable	*B*	SE	Wald	*P*	HR	95%CI	
						LL	UL
Diabetes	0.309	0.154	4.028	0.045	1.361	1.007	1.84
Maximum norepinephrine dose	0.218	0.091	5.795	0.016	1.244	1.041	1.486
Albumin at ICU admission	−0.075	0.015	26.847	< 0.001	0.927	0.901	0.954

### Comparison with the braden scale

**Figure 10:**
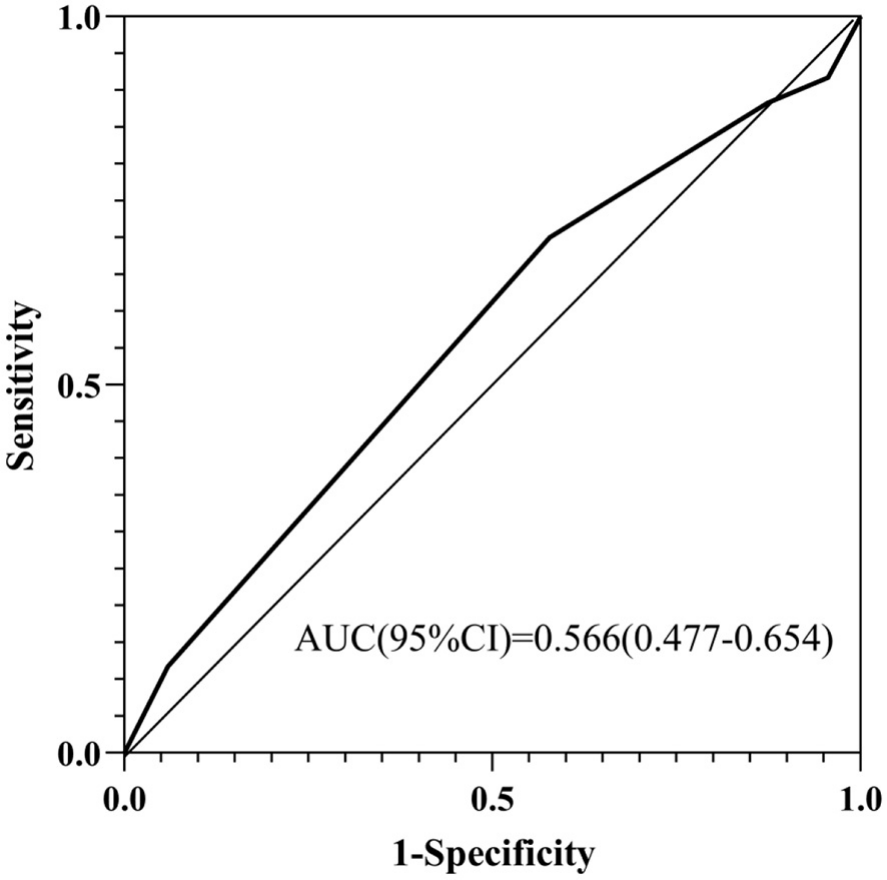
ROC Curve of Admission Braden Score for Predicting Severe PI Progression

We compared the predictive performance of the new nomogram against the standard admission Braden Scale. As shown in Table 7, the Braden score was not an independent predictor of severe PI progression in a multivariable model (OR = 0.837, *P* = 0.303). Its receiver operating characteristic (ROC) curve demonstrated limited discriminatory ability, with an area under the curve (AUC) of 0.566 (95% CI: 0.477–0.654) (Figure 10) may be partly explained by its restricted range of variation (median 10, IQR 10–11), which inherently limits its capacity to stratify risk in this severely ill population. In contrast, the new nomogram achieved substantially higher AUCs of 0.800 (95% CI: 0.736–0.865) in the training set and 0.785 (95% CI: 0.675–0.895) in the validation set (Figure 4), demonstrating its superior accuracy for risk stratification in this specific ICU cohort.

**Table 7:** Multivariable Logistic regression analysis -admission braden score as a predictor of severe PI progression.

Variable	*B*	S.E.	Wald	*P*	OR	95%CI	
						LL	UL
Admission Braden Score	−0.178	0.173	1.059	0.303	0.837	0.596	1.175
Constant	1.054	1.814	0.338	0.561	2.87		

### Subgroup analysis

Subgroup analyses, conducted to assess the robustness of the model's three predictors across clinically relevant strata, revealed heterogeneity in their associations with the outcome. In patients with septic shock (Table 8), diabetes (OR = 4.37, 95% CI 1.58–12.12), maximum norepinephrine dose (OR = 2.07, 95% CI 1.21–3.55), and lower albumin (OR = 0.84, 95% CI 0.78–0.92) all showed significant associations. Conversely, in the non-septic shock subgroup, albumin (OR = 0.80, 95% CI 0.68–0.93) and maximum norepinephrine dose (OR = 2.51, 95% CI 1.07–5.87) remained associated, while diabetes did not (OR=0.91, 95% CI 0.26–3.22). Similarly, in mechanically ventilated patients (Table 9), all three predictors were significant, whereas in the non-ventilated subgroup, only lower albumin showed a statistically significant association (OR = 0.875, 95% CI: 0.652–0.937, *P* = 0.042). Analyses stratified by the site of the most severe PI (Table 10) showed consistent associations for all three predictors in the supine position group. In the lateral/positioning group, diabetes (OR = 3.46, 95% CI 1.04–11.47) and albumin (OR = 0.82, 95%CI 0.66–1.01, *P* = 0.063) were associated. In the device-related group, the limited sample size precluded definitive interpretation. Overall, these results suggest that the strength and significance of the three predictors' associations with PI progression risk may vary depending on the underlying clinical context.

**Table 8:** Multivariable logistic regression analysis for independent predictors of severe pressure injury, stratified by septic shock status.

Variable	*B*	S.E.	Wald	*P*	OR	95%CI	
						LL	UL
Septic shock							
Diabetes (Yes vs. No)	1.476	0.520	8.050	0.005	4.374	1.578	12.121
Maximum norepinephrine dose (μg/kg/min)	0.730	0.273	7.126	0.008	2.074	1.214	3.545
Albumin at ICU admission (g/L)	−0.169	0.043	15.166	< 0.001	0.844	0.775	0.919
Non-septic shock							
Diabetes (Yes vs. No)	−0.092	0.643	0.020	0.887	0.912	0.259	3.215
Maximum norepinephrine dose (μg/kg/min)	0.920	0.433	4.502	0.034	2.509	1.073	5.867
Albumin at ICU admission (g/L)	−0.227	0.079	8.242	0.004	0.797	0.682	0.930

**Table 9:** Multivariable logistic regression analysis for independent predictors of severe pressure injury, stratified by mechanical ventilation status.

Variable	*B*	S.E.	Wald	*P*	OR	95%CI	
						LL	UL
Mechanical ventilation (No)							
Diabetes (Yes vs. No)	0.668	1.006	0.441	0.507	1.951	0.272	14.006
Maximum norepinephrine dose (μg/kg/min)	0.140	0.454	0.096	0.757	1.151	0.473	2.799
Albumin at ICU admission (g/L)	−0.134	0.077	3.035	0.042	0.875	0.652	0.937
Mechanical ventilation (Yes)							
Diabetes (Yes vs. No)	0.852	0.440	3.741	0.043	2.343	1.289	5.553
Maximum norepinephrine dose (μg/kg/min)	0.839	0.264	10.066	0.002	2.314	1.378	3.887
Albumin at ICU admission (g/L)	−0.205	0.044	21.321	< 0.001	0.815	0.747	0.889

**Table 10:** Multivariable logistic regression analysis for independent predictors of severe pressure injury, stratified by most severe pressure injury site.

Variable	*B*	S.E.	Wald	*P*	OR	95%CI	
						LL	UL
Supine position group							
Diabetes (Yes vs. No)	0.960	0.452	4.510	0.034	2.613	1.077	6.338
Maximum norepinephrine dose (μg/kg/min)	0.795	0.273	8.462	0.004	2.215	1.296	3.784
Albumin at ICU admission (g/L)	−0.214	0.046	21.282	< 0.001	0.807	0.737	0.884
Lateral/positioning group							
Diabetes (Yes vs. No)	1.240	0.612	4.107	0.043	3.456	1.042	11.467
Maximum norepinephrine dose (μg/kg/min)	0.338	0.520	0.423	0.516	1.403	0.506	3.890
Albumin at ICU admission (g/L)	−0.204	0.110	3.462	0.063	0.816	0.658	1.011
Device-related group							
Diabetes (Yes vs. No)	0.546	2.260	0.058	0.809	1.726	0.021	144.811
Maximum norepinephrine dose (μg/kg/min)	0.436	1.156	0.142	0.706	1.546	0.161	14.894
Albumin at ICU admission (g/L)	0.063	0.193	0.106	0.745	1.065	0.730	1.554

## Discussion

This study developed and validated a novel nomogram incorporating three readily available clinical factors (diabetes, maximum norepinephrine dose, and serum albumin at ICU admission) to predict the progression from Stage 1 to severe pressure injuries in critically ill patients. The model demonstrated good discrimination, calibration, and clinical utility, providing a practical tool for early risk stratification. Stage 1 PIs represent a pivotal point where timely intervention can prevent irreversible tissue damage. However, not all Stage 1 injuries progress, and current risk assessment tools, such as the Braden Scale, demonstrate limited accuracy in the dynamic ICU environment (12, 13). This often leads to a generalized, inefficient allocation of preventive resources (14, 15). Our model seeks to bridge this gap by facilitating the identification of individuals at highest risk, which could help guide more targeted and intensified preventive strategies.

The independent predictors identified in our model are biologically plausible, each reflecting distinct pathophysiological states that may contribute to the vulnerability to PI progression.

The strong association of hypoalbuminemia with progression risk is consistent with its multifaceted role in critical illness (16). Low serum albumin is associated with a multifaceted deleterious effect on tissue resilience. It can contribute to interstitial edema by compromising plasma oncotic pressure, which in turn may increase local tissue tension and impair microcirculatory flow. Concurrently, its role extends beyond a simple nutritional marker; hypoalbuminemia reflects a state of compromised synthesis and increased catabolism, which could undermine the protein-dependent processes essential for cellular proliferation and tissue repair. Furthermore, persistently low albumin levels serve as an integrated biomarker of both chronic nutritional deficiency and acute systemic inflammation—two conditions frequently intertwined in critically ill patients. The confluence of edema, impaired repair, nutritional deficit, and ongoing inflammation is likely to create a profoundly hostile microenvironment that compromises skin barrier integrity and the capacity for effective wound healing. This pathophysiological cascade could render tissues exquisitely sensitive to pressure-induced ischemia and markedly delay recovery from even minor insults (17, 18).

The link between diabetes and PI progression can be interpreted in the context of its chronic complications. The hallmark microvascular dysfunction can progressively diminish capillary density and reactivity, chronically restricting blood flow and oxygen delivery to the skin, thereby potentially lowering its ischemic threshold. This is compounded by diabetic peripheral neuropathy, which not only blunts the protective pain sensation that normally prompts repositioning but may also dysregulate local neurovascular responses. Additionally, a dysregulated immune response, often present in diabetes, can impair the early recognition of tissue damage and the subsequent inflammatory and repair phases of wound healing. Together, this triad of microangiopathy, neuropathy, and immune dysfunction is thought to create a state of diminished tissue reserve, making the skin less able to withstand pressure, slower to signal distress, and compromised in its ability to mount an effective repair response once injury occurs, which would lower its tolerance to both mechanical stress and ischemia (19, 20).

The maximum norepinephrine dose emerged as a potent prognostic marker, likely signifying the severity of circulatory compromise and its direct impact on cutaneous perfusion. Its elevation signifies a profound state of hemodynamic derangement where endogenous compensatory mechanisms have failed. The administration of norepinephrine at high doses is known to induce an intense, alpha-1 adrenergic receptor-mediated constriction of the peripheral vasculature. This systemic response, while prioritizing blood flow to vital organs, comes at the direct expense of the cutaneous microcirculation. The resulting severe reduction in skin blood flow can create a state of obligatory tissue hypoperfusion, drastically lowering the threshold for pressure-induced ischemia. In this context, even standard supportive surfaces and routine repositioning may be insufficient to prevent injury, as the skin may be likened to functioning in a state of compensated shock, with minimal reserve to withstand additional compressive forces (21, 22).

Our subgroup analyses revealed significant heterogeneity in predictor effects, highlighting the context-dependence of risk and potentially enhancing the model's clinical relevance. Consistent with the profound metabolic and hemodynamic disturbances in critical states, diabetes and a higher maximum norepinephrine dose showed particularly strong associations with progression risk in patients with septic shock, aligning with the known pathophysiology of such high-acuity patients (23, 24). Notably, serum albumin remained a significant protective factor across both septic and non-septic shock subgroups, underscoring its fundamental role in tissue resilience. In the non-septic shock subgroup, the maximum norepinephrine dose and albumin were the key associated factors. This heterogeneity suggests that while a core set of admission markers is informative, the dominant pathophysiological driver of risk-whether metabolic, hemodynamic, or nutritional-may vary with the patient's clinical phenotype, supporting a more nuanced approach to risk assessment and prevention (25, 26).

Our model's performance is comparable to or better than previous prediction tools for hospital-acquired PIs in mixed populations, and it is specifically tailored for the critical transition from Stage 1 to more severe injury. More importantly, it addresses a different and more acute clinical question. Whereas established tools like the Braden Scale help identify patients at risk of developing a PI, our nomogram is specifically tailored to predict which patients with an already present Stage 1 PI are at highest risk for progression. This could support intervention at a critical, potentially reversible juncture, addressing a distinct gap in bedside risk stratification. Notably, in a sensitivity analysis using the more stringent outcome of Stage 3+ or full-thickness injury, the model maintained good discriminatory ability, and hypoalbuminemia remained a significant predictor. This reinforces the potential utility of our core predictors, particularly serum albumin, in forecasting significant tissue damage.

The decision curve analysis provides critical insight into the model's practical utility. The nomogram demonstrated a positive net benefit over the “treat-all” and “treat-none” strategies across a broad range of threshold probabilities (approximately 0.40 to 0.99). This range has direct clinical implications. A threshold probability of 0.40 corresponds to a clinical scenario where a clinician is willing to intensify preventive measures for a patient with an estimated 40% or higher risk of progression-a situation that justifies targeted intervention in resource-aware settings. Conversely, the model's utility extends to an extremely high threshold of 0.99, indicating that it remains valuable even in contexts where interventions are so resource-intensive that they should be reserved only for patients perceived as virtually certain to deteriorate. Thus, the nomogram adds value across a wide spectrum of clinical decision-making, supporting both moderate-risk triage and the identification of the very highest-risk patients.

Several important limitations must be considered when interpreting the findings of this study. Firstly, our model was developed and validated within a single-center, retrospective cohort. This design, while efficient for initial model development, inherently limits the generalizability of our findings to other institutions with different patient populations, nursing protocols, and resource levels. The retrospective design also introduces the possibility of unmeasured confounding from factors not routinely documented in electronic records, such as nuanced differences in turning schedules or the use of specific skin care products. Secondly, the accuracy of the primary outcome hinges on retrospective staging of pressure injuries. Despite adherence to guidelines and a review process for ambiguous cases, we cannot rule out some degree of misclassification, particularly in distinguishing early Stage 1 injuries from other causes of skin redness. This is an inherent constraint of the data source. Third, we recognize that our initial outcome definition, which grouped all injuries of Stage 2 or greater, combines lesions with differing clinical implications. Our subsequent sensitivity analysis, which defined a more severe outcome (Stage 3+), yielded reassuringly consistent results. Nevertheless, the initial broad endpoint may not fully reflect the spectrum of injury severity. Fourth, to serve as an early warning tool, our model intentionally uses only data available at admission. This design choice means it does not incorporate dynamic clinical parameters that change during the ICU stay (e.g., evolving nutritional support or new organ failures), which could potentially improve prediction. Furthermore, the data collection period (2022–2025) coincides with the post-COVID-19 pandemic era. The unique pressures on ICUs during this time regarding patient acuity, staffing, and protocols may influence the model's performance in future, more stable periods. Most importantly, the model has undergone internal validation only. Promising performance in our cohort is a necessary first step, but it is not sufficient. External validation in independent, multi-center populations is an essential prerequisite before any clinical application can be contemplated. Finally, the ultimate test—whether using this nomogram actually improves patient outcomes or is cost-effective—remains a question for future prospective, interventional studies.

## Conclusion

In conclusion, we developed and internally validated a simple nomogram that provides an early estimate of the risk of Stage 1 PI progression. By yielding an individualized risk probability, this tool could inform clinical decision-making regarding the intensity of preventive measures, which might help mitigate the morbidity and costs associated with severe PIs.

## Data Availability

The original contributions presented in the study are included in the article/Supplementary material, further inquiries can be directed to the corresponding author.
