# Supplementary figures and images for: A predictive nomogram for in-ICU deterioration of stage 1 pressure injuries: a retrospective study
Source: Front Med (Lausanne). 2026 May 18;13:1835220. doi: 10.3389/fmed.2026.1835220 (PMC13223033; doi:10.3389/fmed.2026.1835220)

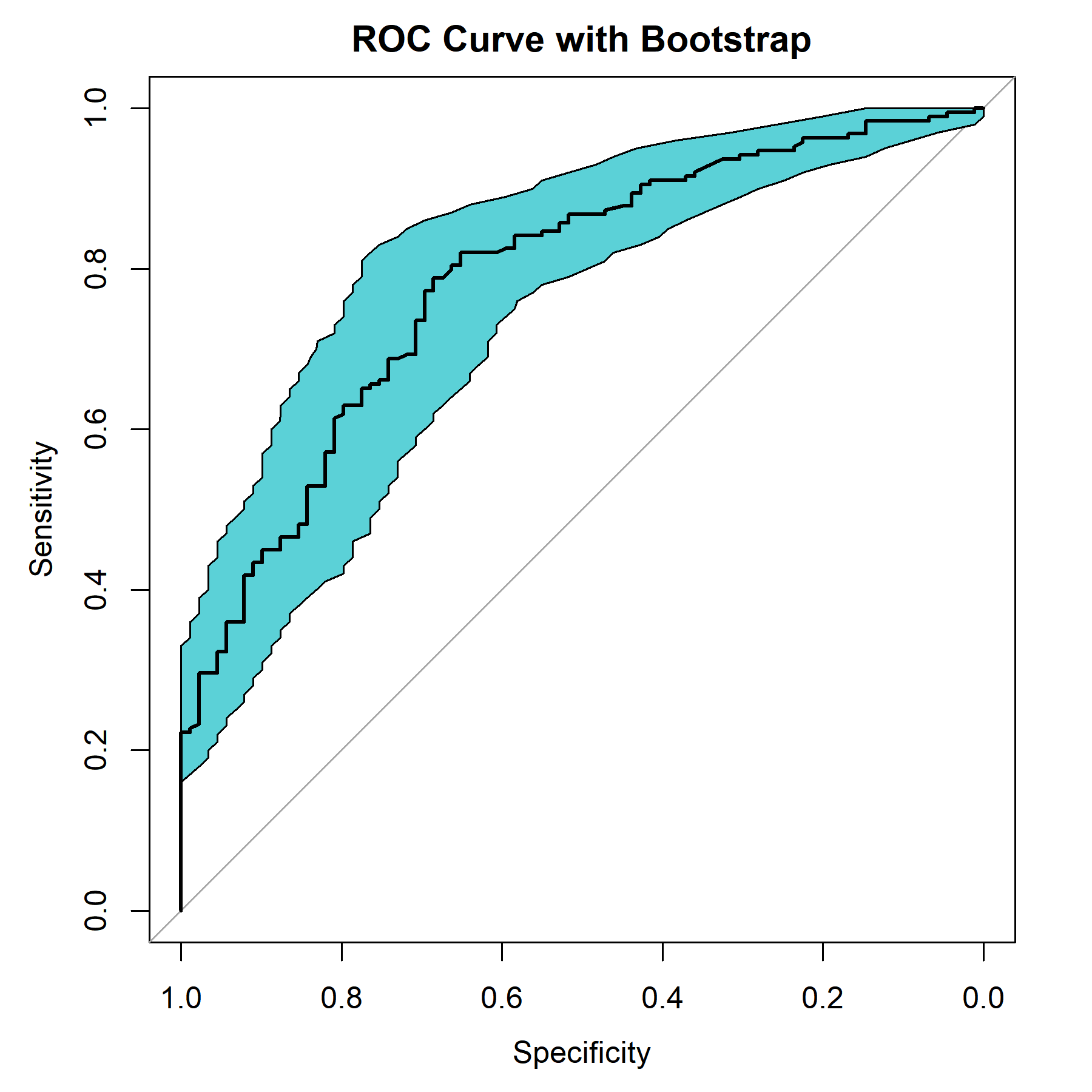

Supplement: Supplementary file 1 [file Image_1.PNG]

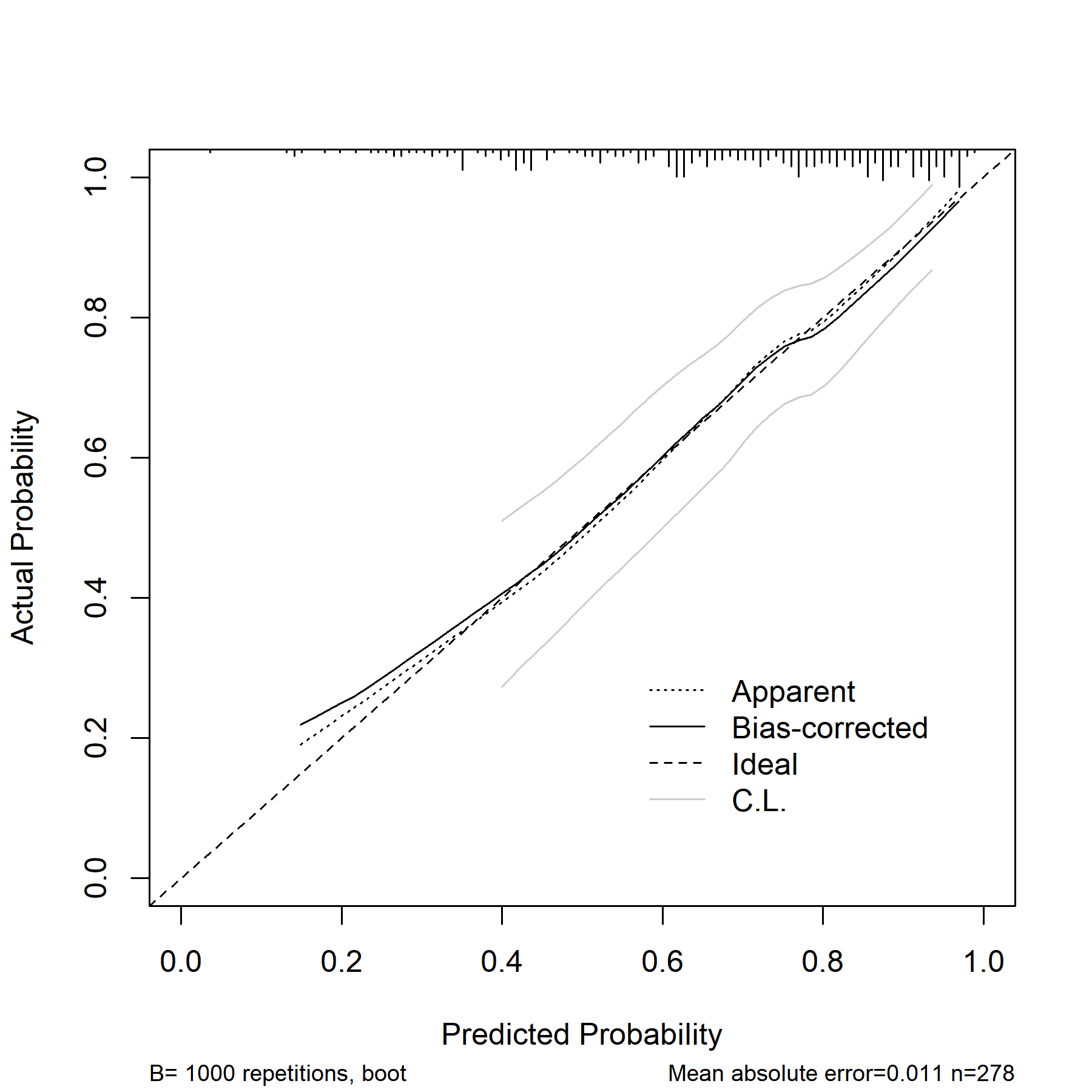

Supplement: Supplementary file 2 [file Image_2.PNG]

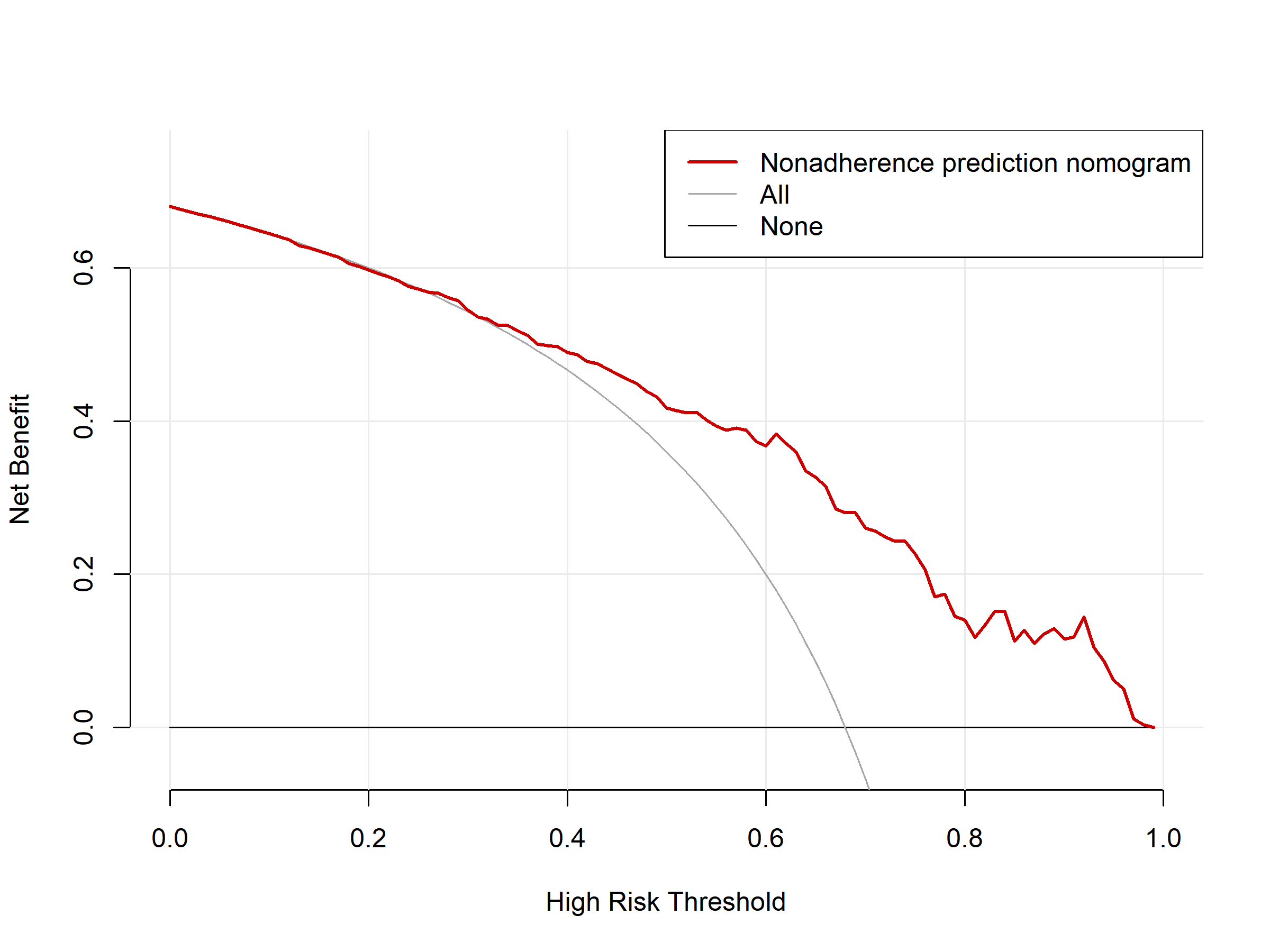

Supplement: Supplementary file 3 [file Image_3.PNG]
