# Supplementary material for: A predictive nomogram for in-ICU deterioration of stage 1 pressure injuries: a retrospective study
Source: Front Med (Lausanne). 2026 May 18;13:1835220. doi: 10.3389/fmed.2026.1835220 (PMC13223033; doi:10.3389/fmed.2026.1835220)
Supplement: Supplementary file 5 [file Table_2.DOCX]

**TRIPOD Checklist**

| **Section/Topic** | **Item No.** | **TRIPOD Checklist Item** | **Reported on Page/Section** | **Details / Remarks** |
| --- | --- | --- | --- | --- |
| **Title and Abstract**​ |  |  |  |  |
| Title | 1 | Identify the study as developing and/or validating a multivariable prediction model, the target population, and the outcome to be predicted. | Title Page | **Yes.**​ The title identifies the study as developing a predictive nomogram for in-ICU deterioration of Stage 1 pressure injuries. |
| Abstract | 2 | Provide a summary of objectives, study design, setting, participants, sample size, predictors, outcome, statistical analysis, results, and conclusions. | Abstract | **Yes.**​ The structured abstract provides all required elements. |
| **Introduction**​ |  |  |  |  |
| Background and objectives | 3a | Explain the medical context (including whether diagnostic or prognostic) and rationale for developing or validating the model, including references to existing models. | Introduction, Paragraph 1-2 | **Yes.**​ The introduction describes the clinical problem of PI progression and the rationale for developing a new predictive tool, citing limitations of existing methods. |
|  | 3b | Specify the objectives, including whether the study describes the development or validation of a model, or both. | Introduction, Paragraph 3 | **Yes.**​ The final paragraph states the objective is to develop and validate a nomogram. |
| **Methods**​ |  |  |  |  |
| Source of data | 4a | Describe the study design or source of data (e.g., randomized trial, cohort, or registry data), separately for the development and validation data sets, if applicable. | Materials and methods, "Study population" | **Yes.**​ Described as a single-center retrospective cohort study. |
|  | 4b | Specify the key dates of the study (start of accrual, end of accrual, and, if applicable, end of follow-up). | Materials and methods, "Study population" | **Yes.**​ Specified as January 2022 to December 2025. |
| Participants | 5a | Specify key elements of the study setting (e.g., level of care, number of participating centers) and inclusion and exclusion criteria. | Materials and methods, "Study population" | **Yes.**​ Single ICU center, inclusion/exclusion criteria clearly listed. |
|  | 5b | Give details of treatments received, if relevant. | Materials and methods, "Data collection" | **Yes.**​ Treatments/interventions (e.g., mechanical ventilation) are listed as candidate predictors. |
| Outcome | 6a | Clearly define the outcome, including how and when assessed. | Materials and methods, "Study population" & "Outcome" | **Yes.**​ Defined as progression from Stage 1 to ≥ Stage 2 PI during ICU stay. |
|  | 6b | Report any actions to blind assessment of the outcome to predictors. | Not applicable | **Not applicable**​ due to retrospective design based on chart documentation. |
| Predictors | 7a | Clearly define all predictors, including how and when they were measured. | Materials and methods, "Data collection" & revised PI assessment section | **Yes.**​ All predictors (demographics, scores, lab values) defined as measured within 24h of ICU admission. PI assessment process detailed. |
|  | 7b | Report any actions to blind assessment of predictors for the outcome. | Not applicable | **Not applicable**​ due to retrospective design. |
| Sample size | 8 | Explain how the study size was arrived at. | Materials and methods, "Statistical analysis" (to be added) | **To be added.**​ Will state: "As an exploratory retrospective study, a formal sample size calculation was not performed a priori. All eligible patients meeting inclusion criteria during the study period were included. The effective sample size and events per variable (EPV) are reported in the Results." |
| Missing data | 9 | Describe how missing data were handled (e.g., complete-case analysis, single imputation, multiple imputation) with details of any imputation method. | Materials and methods, "Study population" & "Statistical analysis" | **Yes.**​ Patients with >20% missing key data were excluded. For other variables with minimal missingness, complete-case analysis was used. |
| Statistical analysis methods | 10a | Describe how predictors were handled in the analyses. | Materials and methods, "Statistical analysis" | **Yes.**​ Describes univariate analysis, LASSO for variable selection, and logistic regression for model building. |
|  | 10b | Specify type of model, all model-building procedures (including any predictor selection), and method for internal validation. | Materials and methods, "Statistical analysis" | **Yes.**​ Logistic regression, LASSO with 10-fold CV, bootstrap validation on training/validation sets. |
|  | 10c | Specify all measures used to assess model performance and, if relevant, to compare multiple models. | Materials and methods, "Statistical analysis" | **Yes.**​ AUC, calibration (slope, intercept, Brier score), and decision curve analysis are specified. |
|  | 10d | Describe any model updating (e.g., recalibration) arising from the validation, if done. | Not done | **No model updating was performed.**​ |
| Risk groups | 11 | Provide details on how risk groups were created, if done. | Not done | **No risk groups were created.**​ The nomogram provides a continuous risk score. |
| **Results**​ |  |  |  |  |
| Participants | 13a | Describe the flow of participants through the study, including the number of participants with and without the outcome and, if applicable, a summary of the follow-up time. A diagram may be helpful. | Results, "Study population" & Figure 1 | **Yes.**​ Participant flow detailed in text and shown in Figure 1. |
|  | 13b | Describe the characteristics of the participants (basic demographics, clinical features, available predictors), including the number of participants with missing data for predictors and outcome. | Results, "Baseline characteristics" & Table 1 | **Yes.**​ Table 1 summarizes characteristics. Missing data handling is stated in Methods. |
| Model development | 14a | Specify the number of participants and outcome events in each analysis. | Results, "Model development and variable selection" | **Yes.**​ Reports n=195 in training set, with 133 events (progressions). |
|  | 14b | If done, report the unadjusted association between each candidate predictor and outcome. | Supplementary Material | **Yes.**​ Univariate analysis results are provided in a supplementary table. |
| Model specification | 15a | Present the full prediction model to allow predictions for individuals (i.e., all regression coefficients, and model intercept or baseline survival at a given time point). | Results, "Multivariable logistic regression" & Table 3 | **Yes.**​ Table 3 provides all coefficients (B), SE, OR, and 95% CI for the final 3-predictor model. |
|  | 15b | Explain how to use the prediction model. | Results, "Nomogram construction" & Figure 3 | **Yes.**​ The nomogram (Figure 3) and its caption provide clear instructions for use. |
| Model performance | 16 | Report performance measures (with CIs) for the prediction model. | Results, "Model Performance and Validation" | **Yes.**​ Reports AUC, sensitivity, specificity, calibration metrics (slope, intercept, Brier) with CIs for both training and validation sets. |
| **Discussion**​ |  |  |  |  |
| Limitations | 18 | Discuss any limitations of the study (such as nonrepresentative sample, few events per predictor, missing data). | Discussion, "Limitations" | **Yes.**​ Discusses single-center retrospective design, potential confounding, and need for external validation. |
| Interpretation | 19a | Give an overall interpretation of the results, considering objectives, limitations, and results from other studies. | Discussion | **Yes.**​ Interprets the findings in clinical context, compares with prior literature, and discusses implications. |
|  | 19b | Discuss the potential clinical use of the model and implications for future research. | Discussion, "Conclusion" | **Yes.**​ Discusses clinical applicability and suggests future validation studies. |
| **Other Information**​ |  |  |  |  |
| Supplementary information | 20 | Provide information about the availability of supplementary resources, such as the study protocol, Web calculator, and data sets. | Not applicable | **Not applicable.**​ No public web calculator or full dataset is provided. |
| Funding | 17 | Give the source of funding and the role of the funders for the present study. | Funding section | **Yes.**​ Funding: This research was funded by the Zhejiang Yangtze River Delta Health Research Fund Project (Fourth Batch) (Grant Number: 2024CSJ-4-B-002) and the Ningbo Major Research and Development Plan Project (Grant Number: 2025Z176). The funders had no role in the study design, execution, analysis, or manuscript preparation. |
