# Supplementary material for: A predictive nomogram for in-ICU deterioration of stage 1 pressure injuries: a retrospective study
Source: Front Med (Lausanne). 2026 May 18;13:1835220. doi: 10.3389/fmed.2026.1835220 (PMC13223033; doi:10.3389/fmed.2026.1835220)
Supplement: Supplementary file 6 [file Table_3.DOCX]

**Supplementary Table S1.** Comparison of baseline characteristics between the training and validation sets.

| Characteristic | Training Set (n=195) | Validation set (n=83) | X²/t/z | *P*-Value |
| --- | --- | --- | --- | --- |
| Outcome variable, n (%) |  |  | 0.014 | 0.904 |
| Non-severe PI | 62(31.79) | 27(32.53) |  |  |
| Severe PI | 133(68.21) | 56(67.47) |  |  |
| Age (years） | 72.51±14.28 | 70.95±16.7 | 0.792 | 0.429 |
| Gender, *n* (%) |  |  | 0.022 | 0.882 |
| Male | 144(73.85) | 62(74.7) |  |  |
| Female | 51(26.15) | 21(25.3) |  |  |
| BMI（kg/m^2^） | 21.8±3.25 | 21.34±3.16 | 1.099 | 0.273 |
| Infection site, *n* (%) |  |  | 6.154 | 0.188 |
| Pulmonary | 133(68.21) | 60(72.29) |  |  |
| Abdominal | 16(8.21) | 5(6.02) |  |  |
| Bloodstream | 0(0) | 2(2.41) |  |  |
| Urinary system | 6(3.08) | 3(3.61) |  |  |
| Others | 40(20.51) | 13(15.66) |  |  |
| Most severe PI site, *n* (%) |  |  | 5.200 | 0.074 |
| Supine position group | 145(74.36) | 54(65.06) |  |  |
| Lateral/positioning group | 42(21.54) | 20(24.1) |  |  |
| Device-related group | 8(4.1) | 9(10.84) |  |  |
| APACHE II at ICU admission | 17(14,22) | 17(12,23) | -0.007 | 0.994 |
| Diabetes, *n* (%) |  |  | 1.691 | 0.193 |
| No | 122(62.56) | 45(54.22) |  |  |
| Yes | 73(37.44) | 38(45.78) |  |  |
| Hospital stay (days) | 23(13,33) | 28(17,36) | -1.544 | 0.123 |
| Lactate (mmol/L) | 1.6(1.3,2.4) | 1.8(1.3,2.6) | -0.964 | 0.335 |
| Septic shock, *n* (%) |  |  | 0.48 | 0.489 |
| No | 126(64.62) | 50(60.24) |  |  |
| Yes | 69(35.38) | 33(39.76) |  |  |
| Vasopressor use, *n* (%) |  |  | 1.801 | 0.18 |
| No | 85(43.59) | 29(34.94) |  |  |
| Yes | 110(56.41) | 54(65.06) |  |  |
| Max norepinephrine dose (µg/kg/min) |  |  | 3.812 | 0.149 |
| <0.1µg/kg/min | 97(49.74) | 40(48.19) |  |  |
| 0.1-0.3µg/kg/min | 42(21.54) | 26(31.33) |  |  |
| >0.3 µg/kg/min | 56(28.72) | 17(20.48) |  |  |
| Mechanical ventilation, *n* (%) |  |  | 1.058 | 0.304 |
| No | 33(16.92) | 10(12.05) |  |  |
| Yes | 162(83.08) | 73(87.95) |  |  |
| Mechanical ventilation duration (days) | 6(3,13) | 6(4,13) | -0.415 | 0.678 |
| Deep sedation, *n* (%) |  |  | 0.195 | 0.659 |
| No | 131(67.18) | 58(69.88) |  |  |
| Yes | 64(32.82) | 25(30.12) |  |  |
| Fluid balance on ICU day 1（ml） | 939(278,1720) | 900(77,1490) | -0.597 | 0.55 |
| Braden score at ICU admission | 10(10,11) | 10(10,11) | -0.509 | 0.611 |
| Braden moisture subscore, *n* (%) |  |  | 1.737 | 0.629 |
| 1 | 1(0.51) | 1(1.20) |  |  |
| 2 | 41(21.03) | 13(15.66) |  |  |
| 3 | 131(67.18) | 57(68.67) |  |  |
| 4 | 22(11.28) | 12(14.46) |  |  |
| Skin moisture management, *n* (%) |  |  | 2.188 | 0.139 |
| No | 149(76.41) | 70(84.34) |  |  |
| Yes | 46(23.59) | 13(15.66) |  |  |
| Avg. enteral nutrition intake (first 3 days) (kcal/day) | 0(0,17.91) | 12(0,18.46) | -0.935 | 0.350 |
| Albumin at ICU admission (g/L) | 28.46±5.83 | 29.31±4.88 | 1.175 | 0.241 |

PI, pressure injury; BMI, body mass index; APACHE II, Acute Physiology and Chronic Health Evaluation II; ICU, intensive care unit.
