# Supplementary material for: A predictive nomogram for in-ICU deterioration of stage 1 pressure injuries: a retrospective study
Source: Front Med (Lausanne). 2026 May 18;13:1835220. doi: 10.3389/fmed.2026.1835220 (PMC13223033; doi:10.3389/fmed.2026.1835220)
Supplement: Supplementary file 7 [file Table_4.DOCX]

**Supplementary Table S2.** Comparison of candidate variables with missing data between the training and validation sets.

| Variable | Training Set | Validation Set | z | P |
| --- | --- | --- | --- | --- |
| APACHE II at ICU admission | 17(14,22) | 17(12,23) | -0.119 | 0.905 |
| Mechanical ventilation duration (days) | 7(3,15) | 6(4,12) | -0.085 | 0.932 |
| Braden score at ICU admission | 10(10,11) | 10(10,11) | -0.109 | 0.913 |
