# Supplementary material for: A predictive nomogram for in-ICU deterioration of stage 1 pressure injuries: a retrospective study
Source: Front Med (Lausanne). 2026 May 18;13:1835220. doi: 10.3389/fmed.2026.1835220 (PMC13223033; doi:10.3389/fmed.2026.1835220)
Supplement: Supplementary file 8 [file Table_5.DOCX]

**Supplementary Table S3.** Sensitivity analysis using Firth’s penalized logistic regression for predictors of severe pressure injury progression.

|  | B | S.E. | Wald | P | OR | 95%CI | |
| --- | --- | --- | --- | --- | --- | --- | --- |
|  |  |  |  |  |  | LL | UL |
| Diabetes | 0.863 | 0.381 | 5.123 | 0.021 | 2.370 | 1.138 | 5.165 |
| Maximum norepinephrine dose (μg/kg/min) | 0.693 | 0.281 | 10.062 | 0.001 | 2.000 | 1.317 | 3.135 |
| Albumin at ICU admission (g/L) | -0.175 | 0.036 | 23.894 | ＜0.001 | 0.840 | 0.779 | 0.898 |
| Constant | 4.406 | 1.086 | 16.458 | ＜0.001 |  |  |  |
